# Latency-Associated Viral Interleukin-10 (IL-10) Encoded by Human Cytomegalovirus Modulates Cellular IL-10 and CCL8 Secretion during Latent Infection through Changes in the Cellular MicroRNA hsa-miR-92a

**DOI:** 10.1128/JVI.02424-14

**Published:** 2014-12

**Authors:** Emma Poole, Selmir Avdic, Jemima Hodkinson, Sarah Jackson, Mark Wills, Barry Slobedman, John Sinclair

**Affiliations:** a Department of Medicine, University of Cambridge, Addenbrooke's Hospital, Cambridge, United Kingdom; b Human Cytomegalovirus Research Group, Discipline of Infectious Diseases and Immunology, University of Sydney Camperdown, Camperdown, NSW, Australia

## Abstract

The UL111A gene of human cytomegalovirus encodes a viral homologue of the cellular immunomodulatory cytokine interleukin 10 (cIL-10), which, due to alternative splicing, results in expression of two isoforms designated LAcmvIL-10 (expressed during both lytic and latent infection) and cmvIL-10 (identified only during lytic infection). We have analyzed the functions of LAcmvIL-10 during latent infection of primary myeloid progenitor cells and found that LAcmvIL-10 is responsible, at least in part, for the known increase in secretion of cellular IL-10 and CCL8 in the secretomes of latently infected cells. This latency-associated increase in CCL8 expression results from a concomitant LAcmvIL-10-mediated suppression of the expression of the cellular microRNA (miRNA) hsa-miR-92a, which targets CCL8 directly. Taking the data together, we show that the previously observed downregulation of hsa-miR-92a and upregulation of CCL8 during HCMV latent infection of myeloid cells are intimately linked via the latency-associated expression of LAcmvIL-10.

**IMPORTANCE** HCMV latency causes significant morbidity and mortality in immunocompromised individuals, yet HCMV is carried silently (latently) in 50 to 90% of the population. Understanding how HCMV maintains infection for the lifetime of an infected individual is critical for the treatment of immunocompromised individuals suffering with disease as a result of HCMV. In this study, we analyze one of the proteins that are expressed during the “latent” phase of HCMV, LAcmvIL-10, and find that the expression of the gene modulates the microenvironment of the infected cell, leading to evasion of the immune system.

## INTRODUCTION

The species-specific human cytomegalovirus (HCMV) is widespread within the human population: estimates of seroprevalence vary between 50% and 90%, depending on socioeconomic factors (1). In healthy individuals, a robust T cell response to primary infection is believed to ensure that clinical symptoms rarely occur. However, like all herpesviruses, HCMV persists for life, and this is mediated, at least in part, by its ability to enter a latent life cycle (2, 3).

In general, latent infection of otherwise healthy carriers is also asymptomatic, as is the sporadic reactivation of the virus, which is believed to occur routinely in latently infected individuals *in vivo*. However, in immunocompromised individuals (including AIDS patients and transplant recipients), primary infection, as well as reactivation from latency, carries a risk of severe morbidity and mortality (2, 3).

The differences between lytic and latent infection with HCMV are striking. Lytic infection is initiated by the expression of virus-encoded immediate-early (IE) genes; the subsequent expression of viral early (often viral DNA replication-associated) and late (often viral packaging-associated) genes is followed by the release of infectious virions. A number of cell types support lytic infection, including fibroblasts and endothelial cells, and this occurs over 48 to 72 h *in vitro* (4). Conversely, in certain cell types, such as undifferentiated monocytes and their CD34^+^ progenitors, latent infection can be established. During latency, the lytic transcription program is heavily suppressed. This results in a lack of production of infectious virions, and viral gene expression is restricted to a substantially reduced number of latency-associated transcripts (2, 3, 5–11). However, these latently infected cells can reactivate virus as a result of their differentiation into macrophages and dendritic cells (DCs) (2, 3).

Despite a profoundly limited viral transcription profile, a substantial number of changes in cellular gene expression are known to occur during latent infection of myeloid cells with HCMV (12–14), including changes to cellular microRNAs (miRNAs) (15), as well as changes to the cellular secretome (16, 17). For example, in latently infected CD34^+^ cells, the upregulation of the cellular chemokine CCL8 acts as a CD4^+^ T cell chemoattractant, but concomitant increases in latency-associated secretion of other immunomodulatory factors, such as transforming growth factor β (TGF-β) and cellular interleukin 10 (cIL-10), neutralizes the effector functions of these recruited T cells (16). Although latency-associated regulation of the cellular miRNA hsa-miR-92a has been implicated in the control of cIL-10 expression during latency (15), how latent infection modulates expression of, e.g., CCL8 is far from clear.

Intriguingly, although latent HCMV infection robustly induces expression of cIL-10, the virus also encodes IL-10 homologues. Two isoforms of virus-encoded IL-10 are generated by alternative splicing from the viral UL111A gene. One of these is a protein of 175 amino acids, termed cmvIL-10, which is expressed during lytic infection and has the expression kinetics of a late gene (18–20). The second isoform, predicted to consist of 139 amino acids and termed LAcmvIL-10, has a C-terminal truncation and is expressed during both lytic and latent infection (11, 21–23). HCMV is not the only CMV that encodes a cellular IL-10 homologue. An IL-10 homologue encoded by the UL111A open reading frame (ORF) has also been identified in rhesus macaque CMV (RhCMV). Although it has a slightly different gene structure than cmvIL-10, like cmvIL-10, it shows low amino acid identity to host cellular IL-10 (20).

Although the full-length cmvIL-10 gene has only 27% amino acid identity to the human cIL-10 gene, cmvIL-10 has a number of functions in common with cIL-10: it forms homodimers and binds the cIL-10 receptor (24); it triggers STAT3 phosphorylation and activation of the JAK/STAT signaling pathway (23, 25); it signals via the phosphoinositide-3-kinase pathway, contributing to cytokine suppression (26); and it shares the ability of cIL-10 to prevent NF-κB activity via inhibition of IKK (26).

In contrast, LAcmvIL-10 appears quite dissimilar to cIL-10 and cmvIL-10. Although, like cIL-10 and cmvIL-10, it can downregulate major histocompatibility complex (MHC) class II in latently infected granulocyte/macrophage progenitors (GMPs) (11, 22), it either does not signal through the IL-10 receptor (IL-10R) or it engages the receptor in a different way than cIL-10 and cmvIL-10 (23). Consequently, there is a dearth of knowledge regarding the functions of LAcmvIL-10 during latency.

We now show that, during latent infection of myeloid cells, LAcmvIL-10 mediates the known latency-associated increase in secreted cellular CCL8 and that this is due to concomitant suppression by LAcmvIL-10 of expression of the cellular miRNA hsa-miR-92a, which in itself contributes to a number of effects during latency (15), as well as targeting CCL8 directly.

## MATERIALS AND METHODS

### Cells and viruses.

CD34^+^ hematopoietic progenitor cells from the peripheral blood of granulocyte colony-stimulating factor (G-CSF)-mobilized donors were resuscitated as described by the manufacturer (Lonza) and maintained in X-vivo 15 (Lonza) in the absence of serum or growth factors. Primary CD14^+^ monocytes were isolated from venous blood, as described previously (27). The purity of isolated monocytes was determined by flow cytometric detection of CD14^+^ cells, resulting in mean CD14^+^ populations of 98.1%. The Merlin bacterial artificial chromosome (BAC) (28) was used to generate the UL111A deletion virus and the revertant, as previously described (29).

### Proteins.

Recombinant LAcmvIL-10 and the control protein were generated as described previously (22).

### qPCR and RT-qPCR.

For the quantification of hsa-miR-92a, the Cell-CT reverse transcription-quantitative PCR (RT-qPCR) kit (Ambion) was used with specific primers and TaqMan probes to hsa-miR-206, and hsa-miR-92a was analyzed relative to the housekeeping miRNA hsa-miR-16 using standard parameters, as described previously (15).

For the quantification of IE transcript, RNA was isolated using the miRNeasy minikit and amplified using the Quantitect virus +ROX virus kit (Qiagen). The IE and UL138 (gene 138 of the unique long viral-genome region) transcripts were amplified alongside the glyceraldehyde-3-phosphate dehydrogenase (GAPDH) housekeeping transcript using the following primers and probes: IE, CAAGAACTCAGCCTTCCCTAAGAC and TGAGGCAAGTTCT**GC**AATGC with the probe (FAM [6-carboxyfluorescein])CCAATGGCTGCAGTCAGGCCATG(TAMRA [6-carboxytetramethylrhodamine; Sigma]), and GAPDH, GGAAGCTTGTCATCAATG and CCCCACTTGATTTTGGAG with the probe (JOE [Sigma])ATCACCATCTTCCAGGAGCGAG(BHQ1 [black hole quencher 1]). The viral transcript UL138 was detected with primers CGCTGTTTCTCTGGTTAG and CAGACGATACCGTTTCTC with the probe (Cy5)CCGACGACGAAGACGATGAAC(BHQ2). Samples were analyzed and processed with an ABI 7500 Fast Real Time machine using MicroAmp Fast Optical 96-well reaction plates with the following RT parameters: 50°C for 20 min, followed by heat inactivation at 95°C for 5 min and then the PCR steps (50 cycles of 95°C for 15 s and 60°C for 45 s).

Virus genome quantification was carried out using a qPCR as described previously (30), modified to remove the preamplification PCR step.

The quantification of RT-qPCR products was performed either by the calculation of ΔΔ*C_T_* values as described previously (15) or by a direct determination of HCMV copy numbers from the WHO HCMV standard control curve, as previously described (30).

### Semiquantitative PCR.

For the validation of latency, RNA was isolated from CD34^+^ cells using TRIzol (Invitrogen), and RT-PCRs were performed for IE72 (IE), UL138, and GAPDH for 20 cycles of amplification under previously published conditions (15, 21). Cellular IL-10 was quantified using primers 5′-GCCTAACATGCTTCGAGATC and 5′-TGATGTCTGGGTCTTGGTTC after RNA was extracted using an Analytik Jena RNA kit and first-strand cDNA amplified using an Agilent Affinity Script qPCR cDNA synthesis kit, and a qPCR was run with 2× Brillina II SYBR Green qPCR master mix (Agilent Technologies) at 95°C for 10 min and then 40 cycles consisting of 95°C for 15 seconds and 60°C for 45 s.

### Fluorescence-activated cell sorting (FACS).

Monocytes were treated with 50 ng/ml of recombinant LAcmvIL-10 or a control protein for 24 h. The treated monocytes were harvested and then stained with HLA-DR allophycocyanin (APC) antibody (BD Biosciences). Following fixation, the samples were acquired on a FACSCalibur (BD Biosciences) and analyzed using Flowjo software (TreeStar Inc.).

### Enzyme-linked immunosorbent assay (ELISA).

For CCL8, supernatants were quantified using the RayBiotech kit. For IL-10, supernatants were assayed using the Quantikine kit (R&D Systems).

### Cell viability assay.

Cells were stained with trypan blue, and the blue cells were enumerated.

## RESULTS

### Validation of the UL111A deletion mutant virus during HCMV latency in primary myeloid cells.

It has previously been reported that HCMV expresses two different isoforms of viral IL-10 (vIL-10), resulting from differential splicing, which have been designated cmvIL-10 and LAcmvIL-10 (11, 19, 20). LAcmvIL-10 is known to be expressed during both HCMV lytic and latent infection (11, 21). However, to date, expression of cmvIL-10 has been described only during lytic infection.

**FIG 1:**
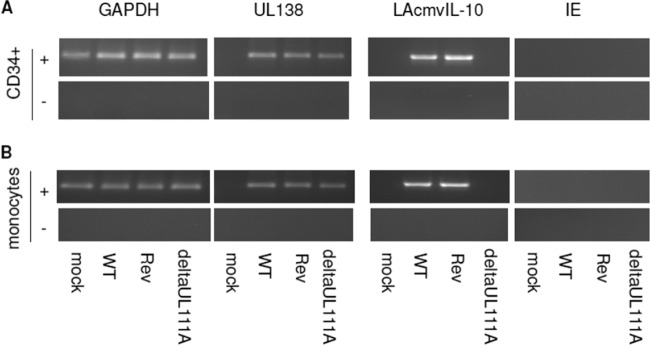
LAcmvIL-10 is transcribed during experimental latency in both CD34^+^ cells and CD14^+^ monocytes. CD34^+^ (A) or CD14^+^ (B) cells were latently infected with HCMV for 9 days and then harvested for GAPDH, UL138, LAcmvIL-10, or IE mRNA as indicated and analyzed by RT-PCR in the presence (+) or absence (−) of reverse transcriptase enzyme. Mock, mock infected; Rev, revertant.

In order to test the role of LAcmvIL-10 during latent infection, we generated a recombinant virus in which the complete UL111A open reading frame had been deleted. A UL111A deletion virus has been previously characterized (31), but it was based on the extensively passaged AD169 laboratory isolate of HCMV devoid of the UL-b′ region of the genome, which is known to carry a number of viral genes important for immune evasion and latent infection (8, 32–35). Consequently, we generated the equivalent UL111A deletion in the clinical isolate Merlin and tested its ability to undergo latent infection in primary myeloid cells. Figure 1A shows that, after experimental latent infection of CD34^+^ cells, both wild-type (WT) virus and the UL111A deletion mutant virus (deltaUL111A) expressed the latency-associated viral UL138 RNA in the absence of viral IE transcription, consistent with latent infection in these cells. LAcmvIL-10 was also detected in CD34^+^ cells latently infected with WT or revertant virus but, as expected, was not detected after latent infection with the deltaUL111A virus. We also confirmed these observations in a second model of HCMV latency. Figure 1B shows that CD14^+^ monocytes latently infected with WT or revertant virus also expressed both UL138 and LAcmvIL-10, but no LAcmvIL-10 was detectable in these cells latently infected with deltaUL111A virus.

### deltaUL111A virus is less efficient at establishing latency in CD34^+^ myeloid progenitors and CD14^+^ monocytes.

Previous studies, using an AD169 deltaUL111A virus, have shown little difference in the ability of this vIL-10 deletion mutant to undergo latent infection in GMPs (36).

**FIG 2:**
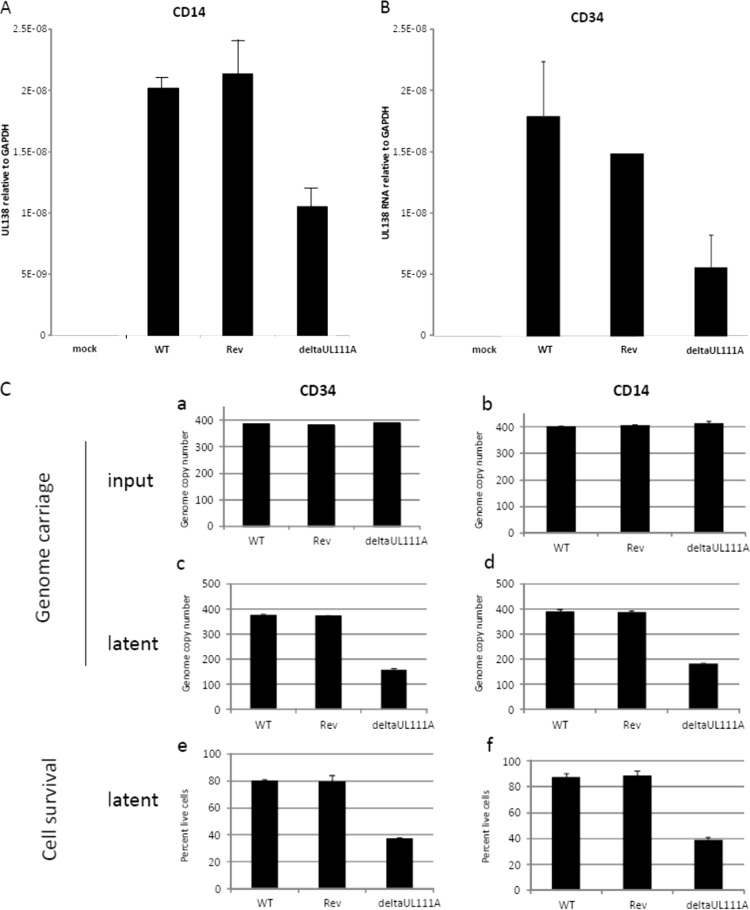
HCMV lacking UL111A has decreased capacity for establishing latency. (A and B) Primary CD14^+^ or CD34^+^ monocytes were latently infected with WT, revertant, or deltaUL111A HCMV for 10 days and analyzed for UL138 RNA levels by qRT-PCR. The graphs represent ΔΔ*C_T_* values from two experiments. (C) (a to d) Alternatively, cells were lysed for DNA extraction after infection to assess genome input (a and b) or after the establishment of latency at 10 days (c and d). (e and f) After 10 days, the cells were also stained with trypan blue to assess cell survival. The data represent two biological replicates of triplicate samples plus standard deviations.

In contrast, using the Merlin clinical isolate, the lack of UL111A did show a decrease in the establishment of latent infection of CD14^+^ monocytes, as well as in latently infected CD34^+^ cells. In these cells, the levels of latency-associated expression of UL138 were qualitatively lower in both CD34^+^ and CD14^+^ monocytes when cells were latently infected with the deltaUL111A virus than when they were infected with either the WT or revertant (Fig. 1A and B), which was confirmed quantitatively by RT-qPCR (Fig. 2A and B). These analyses suggest that the establishment of latency with clinical isolates of HCMV is less efficient in the absence of LAcmvIL-10 in these myeloid cell types.

In order to determine why deltaUL111A virus appeared to show a reduced efficiency of latent carriage, at least on the basis of levels of latent expression of UL138, we quantified the efficiency of genome carriage in cells latently infected with wild-type or deltaUL111A virus. Figure 2C (graphs a and b, respectively) show that, although the number of input genomes used to infect CD34^+^ and CD14^+^ cells were equivalent for all virus isolates, the number of deltaUL111A virus genomes carried by both cell types after 10 days of latency was clearly reduced compared to the wild-type or revertant virus (Fig. 2C, graphs c and d). To determine whether this was due to differential cell death, latently infected cells were analyzed by trypan blue exclusion. Consistent with the decrease in genome carriage, cells latently infected with deltaUL111A virus showed increased levels of cell death compared to either wild-type or revertant virus in both CD34^+^ and CD14^+^ progenitor cells (graphs e and f, respectively). These data demonstrate that LAcmvIL-10 is likely to play a role in enhancing cell survival, a property it may share with cIL-10, which is known to increase cell survival in myeloid progenitors generally and during HCMV latency (15).

### Recombinant LAcmvIL10 is functionally active.

**FIG 3:**
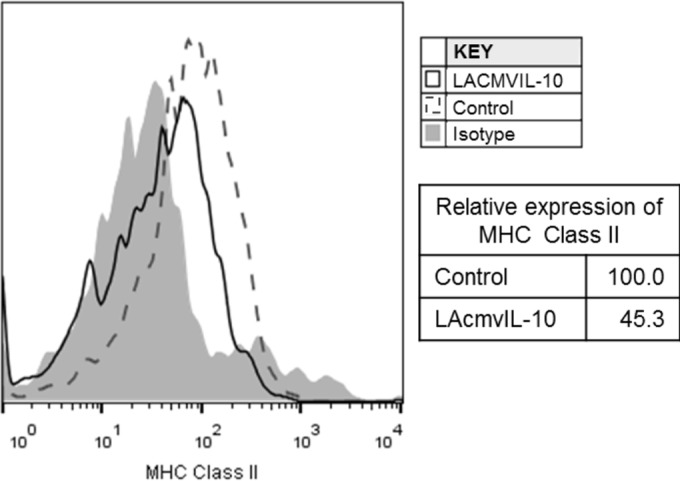
LAcmvIL-10 causes downregulation of MHC class II. CD14^+^ monocytes were incubated in the presence of recombinant LAcmvIL10 or control protein at 50 ng/ml for 24 h before fixing and staining for HLA-DR and analysis by FACS.

To assess the potential role of LAcmvIL-10 in the maintenance of latent viral genomes, a recombinant bacterially expressed LAcmvIL-10 protein was generated, as previously described (22). To confirm the functionality of the proteins, we first analyzed their abilities to downregulate MHC class II; both the LAcmvIL-10 and cmvIL-10 proteins have been shown to reduce MHC class II cell surface expression on treated GMPs (22). Figure 3 shows that, as expected, treatment of CD14^+^ monocytes with LAcmvIL-10 resulted in decreased expression of MHC class II, confirming the functionality of the purified recombinant LAcmvIL-10.

### Recombinant LAcmvIL-10 upregulates cellular IL-10 via a decrease in cellular hsa-miRNA-92a.

**FIG 4:**
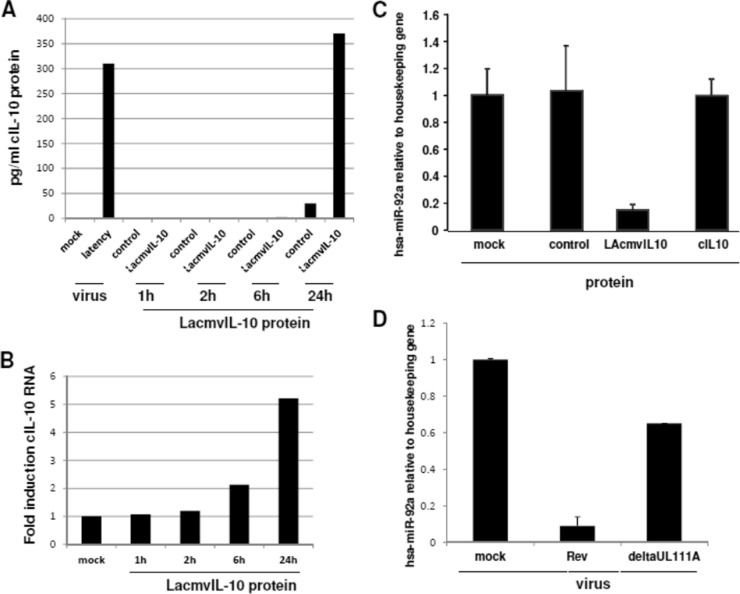
Recombinant LAcmvIL-10 causes cIL-10 upregulation and hsa-miR-92a downregulation. CD14^+^ cells were treated with the LAcmvIL-10 protein (A, B, and C) or infected with HCMV deltaUL111a virus or the revertant (A and D), and then supernatants from cells treated with the indicated recombinant proteins were assayed for levels of cellular IL-10 by ELISA (A) or harvested for cIL-10 RNA quantitation (B) or cells were harvested and assayed for levels of hsa-miR-92a by RT-qPCR (C and D). The data represent two biological replicates of duplicate (A) or triplicate (B, C, and D) samples. The error bars indicate standard deviations.

Latent infection of CD34^+^ progenitors with the Merlin isolate of HCMV is known to result in an increase in secreted cIL-10. This is an important survival factor for primary myeloid progenitor cells, such as CD34 and CD14^+^ cells (15, 37–39). Furthermore, since cIL-10 is known to autoregulate its own expression, we reasoned that at least one function of latency-associated expression of viral IL-10 could be to help modulate cIL-10 expression. Consequently, we tested whether the LAcmvIL-10 protein had any effect on the expression of cIL-10 in myeloid cells. Figure 4A shows that, as expected, latent infection of CD14^+^ cells results in increased secretion of cellular IL-10, and interestingly, treatment of CD14^+^ cells with recombinant LAcmvIL-10 also led to time-dependent induction of cIL-10. These results were verified at the transcription level, which again indicated concomitant increases in levels of cIL-10 transcript upon LAcmvIL-10 treatment (Fig. 4B).

Our previous work has also shown that the latency-associated increase in cellular IL-10 results, at least in part, from a latency-associated decrease in the cellular miRNA hsa-miR-92a (15). However, how latent infection affects this decrease in hsa-miR-92a is unclear. As LAcmvIL-10 increased cIL-10 expression, we reasoned that this effect might also be mediated through an effect of LAcmvIL-10 on hsa-miR-92a. Consequently, we tested whether treatment of myeloid cells with the LAcmvIL-10 protein altered expression of hsa-miR-92a. Figure 4C shows that LAcmvIL-10 protein clearly reduced the levels of hsa-miR-92a RNA compared to controls or the cIL-10 protein. Consistent with this, when latency was established in CD14^+^ cells using the deltaUL111A virus, the latency-associated decrease in hsa-miR-92a was abrogated compared to revertant virus (Fig. 4D).

These data suggest, therefore, that LAcmvIL-10 may be a significant contributing factor to the latency-associated decrease in cellular hsa-miR-92a and the concomitant increase in expression of cIL-10 that we have previously observed upon latent infection of CD34^+^ cells (15, 16).

### The downregulation of hsa-miR-92a by LAcmvIL-10 also causes CCL8 upregulation due to direct targeting of CCL8 by hsa-miR-92a.

**FIG 5:**
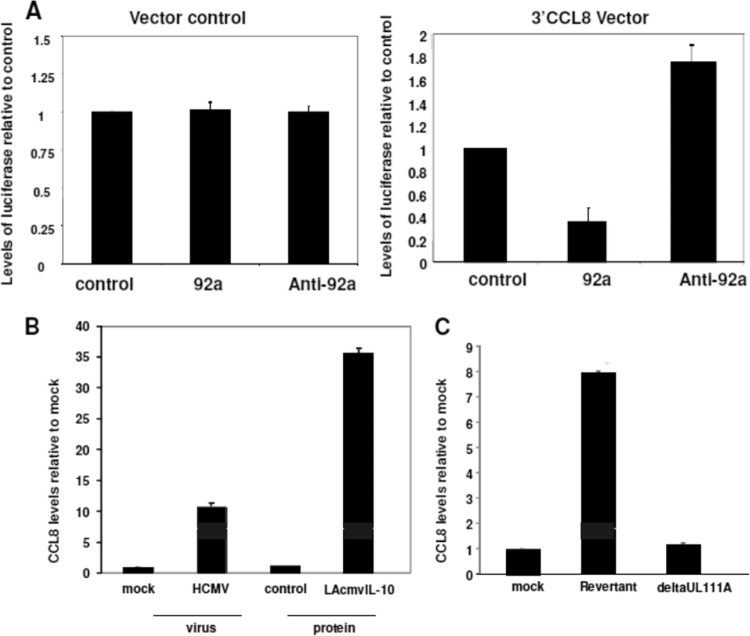
hsa-miR-92a targets CCL8, and LAcmvIL-10 causes CCL8 upregulation. (A) CD14^+^ cells were transfected with an empty luciferase vector (left graph) or with a luciferase vector containing the 3′ UTR of CCL8 (3′CCL8) (right graph) in the presence or absence of hsa-miR-92a mimic (92a), as well as with an hsa-miR-92a antagomir (Anti-92a), as indicated, and luciferase levels were plotted relative to untreated controls. (B and C) CD14^+^ cells were also treated with the indicated recombinant proteins and then assayed for levels of secreted CCL8 by ELISA (B), or CD14^+^ cells were infected with either the viral IL-10 deletion virus (deltaUL111A) or a revertant virus, and following the establishment of latency, the supernatants were assayed for levels of CCL8 by ELISA (C). The data represent two biological replicates of triplicate (A and B) or duplicate (C and D) samples. The error bars indicate standard deviations.

We have previously shown that, in addition to the upregulation of cIL-10 in the secretomes of latently infected cells, there is also an increase in secretion of the cellular chemokine CCL8 (16). Interestingly, CCL8 is a predicted target of hsa-miR-92a using computer prediction algorithms (such as EBI [http://www.ebi.ac.uk/enright-srv/microcosm/htdocs/targets/v5/] and Targetscan [http://www.targetscan.org]). To confirm that CCL8 mRNA is directly targeted by hsa-miR-92a, we carried out luciferase-based assays in which the predicted target of hsa-miR-92a in the 3′ untranslated region (UTR) of CCL8 was cloned into a luciferase expression vector and assayed for levels of luciferase expression after cotransfection with an hsa-miR-92a mimic. Figure 5A (right graph) shows that hsa-miR-92a was indeed able to decrease luciferase expression, and conversely, an antagomir to hsa-miR-92a led to an increase in levels of luciferase. In contrast, hsa-miR-92a did not suppress luciferase expression from the parental luciferase vector (Fig. 5A, left graph).

We next tested whether LAcmvIL-10 was able to affect levels of endogenous cellular CCL8 expression in myeloid primary cells. Figure 5B shows that, as expected, latent infection of CD14^+^ monocytes resulted in an increase in secreted CCL8, similar to our previous observations in latently infected CD34^+^ cells (16). Treatment of CD14^+^ monocytes with recombinant LAcmvIL-10 also resulted in a substantial induction of CCL8 expression (Fig. 5B). The levels of CCL8 induced by LAcmvIL-10 were also much higher than those in latently infected cells, suggesting, perhaps, that much lower levels of LAcmvIL-10 are actually expressed by latently infected cells.

Consistent with these observations, when latency was established using the deltaUL111A virus, no such induction of CCL8 was observed (Fig. 5C). This argues that LAcmvIL-10 is the primary regulator of CCL8 induction during HCMV latency.

## DISCUSSION

Previous work has underscored the importance of the regulation of the microenvironment around cells latently infected with HCMV; it helps cell survival, as well as aiding immune evasion (15–17). Although some of the mechanisms by which latent viruses mediate such changes are starting to be addressed (8, 15, 16, 40–43), a more detailed understanding of how latency-associated changes impact the basic biological properties of the cell, to the advantage of latent virus, and how they are mediated by latent infection is crucial.

Although there are conflicting reports on the exact spectrum of viral gene expression during HCMV latency in the myeloid lineage (2, 3, 5–11), it is clear that, in comparison to the lytic transcription program, the latency-associated transcriptome is heavily suppressed (2, 3, 44, 45). Expression of a number of viral genes has been confirmed during natural latency, and latency-associated functions for some of them (8, 40, 46), including LAcmvIL-10 (41, 43), have been posited.

Most reported functions of HCMV-encoded IL-10 have described the effects of cmvIL-10. They include the inhibition of secretion of proinflammatory cytokines by lipopolysaccharide (LPS)-stimulated peripheral blood mononuclear cells (PBMCs), monocytes, monocyte-derived DCs (MDDCs), and plasmacytoid DCs (PDCs) (18, 21, 25, 41, 47); the stimulation of DC migration toward peripheral lymph nodes (18); and the stimulation of B cells (23). Most pertinent to our study, cmvIL-10 can induce cIL-10 expression.

A number of studies have also directly compared the functions of cmvIL-10 with those of LAcmvIL-10. Most have observed that cmvIL-10 shares more functions with cIL-10 than LAcmvIL-10 does. For example, as observed for cIL-10, cmvIL-10, but not LAcmvIL-10, increases the expression of the fragment crystallizable IgG (FCγ) receptors CD32 and CD64, as well as FCγ receptor-mediated phagocytosis (48). Similarly, like cIL-10, cmvIL-10 inhibits the expression of proinflammatory cytokines in LPS-stimulated MDDCs, whereas LAcmvIL-10 does not (21, 22).

One function common to cmvIL-10 and LAcmvIL-10 is the ability of both recombinant proteins to downregulate MHC class II *in vitro* (Fig. 3). However, there have been fewer studies analyzing the effects of LAcmvIL-10 in the context of latent infection. Previous studies suggested that LAcmvIL-10 decreases the differentiation of CD34^+^ progenitors into DCs (43), as when latency was established with a virus lacking UL111A, the expression of cytokines associated with DC formation was increased, and consequently, the number of myeloid DCs in the population was also increased (43). Our study now shows that LAcmvIL-10 plays a role in regulating cellular miRNA expression and that this affects the levels of specific secreted cellular proteins.

HCMV is not the only herpesvirus to express a cIL-10 homologue. Virus-encoded homologues of cIL-10 are present throughout the family Herpesviridae and emphasize the importance of the cytokine during virus infection (49). It is interesting that the gammaherpesvirus Epstein-Barr virus (EBV) expresses a homologue of cIL-10 (EBVIL-10) that has a much higher sequence homology to cIL-10 than HCMV-encoded IL-10 does. Functionally, EBVIL-10 mimics many of the immune-suppressive properties of cIL-10 (50). Clearly, the expression of such an immune-suppressive cytokine, which would impart clear advantages for the virus, argues that there is a requirement for common functions of this viral IL-10 during both virus life cycles. However, the fact that HCMV also expresses an IL-10 isoform specific to latent infection (LAcmvIL-10) suggests that certain additional functions not common to cmvIL-10 are required specifically during latency.

As previously described for HCMV latency (15), EBV is also known to alter the cellular miRNAome, and in particular the cellular 17-92 miRNA cluster, to affect the transcription of viral gene products (51). However, the mechanism by which the cellular 17-92 miRNA cluster is regulated by latent EBV infection has not been determined. Our observations now describe a novel role for LAcmvIL-10 in the manipulation of cellular hsa-miR-92a, part of the 17-92 miRNA cluster, during latent HCMV infection, which in part explains the ability of HCMV to modulate the latency-associated expression of two important secreted cellular proteins, cIL-10 and CCL8 (16). The mechanism by which LAcmvIL-10 regulates hsa-miR-92a is not clear. Combinations of transcription factors, such as p53, c-myc, DNMt1, DNMT3b, and REST, have been shown to be important for the regulation of the transcription of a number of cellular miRNAs (52, 53). However, we do know that other members of the 17-92 miRNA cluster are not concomitantly downregulated during latent HCMV infection (15). Since the whole cluster is likely to be regulated by the same promoter (54), the mechanism by which LAcmvIL-10 specifically regulates the levels of hsa-miR-92a are unclear. Similarly, whether LAcmvIL-10 also mediates any of the other latency-associated changes in cellular miRNAs we have previously identified (15) is not known but is under investigation.

It is becoming clear that, as well as targeting active lytic infection, targeting latent HCMV infection will be important to reduce disease in a number of clinical settings (46). To date, all active antiviral therapies for HCMV target lytic infection and so, by design, do not target latently infected cells. A proof of principle that changes in latently infected cells could become targets for novel antiviral therapies has recently been described (46), and it will become increasingly important to expand such “latency-targeting” strategies.

Our view is that LAcmvIL-10, by enhancing cell survival, impacts latent HCMV infection and carriage of latent genomes, as reported here, as well as in a previously published report suggesting that LAcmvIL-10 plays a role in myeloid differentiation (43).

Our experiments do not distinguish between paracrine and autocrine effects of LAcmv-IL-10. It is possible that prolife signals resulting in enhanced maintenance of the viral genome in latently infected CD14^+^ and CD34^+^ cells result from the effects of secreted LAcmvIL-10 on bystander cells, which then impart a microenvironment conducive to latent carriage. Alternatively, secreted LAcmvIL-10 may act directly on the latently infected cell. Regardless of the mechanism (paracrine, autocrine, or both), the fact that cells latently infected with HCMV in the absence of LAcmvIL-10 are less able to maintain the latent genome argues for biological relevance.

Consequently, LAcmvIL-10 could represent a viable therapeutic target. In contrast to EBVIL-10, which has a high degree of amino acid identity to cIL-10 (49), LAcmvIL-10 has much reduced homology. Consequently, it may lend itself more easily to the generation of LAcmvIL-10-specific therapeutic antibodies with little cross-reactivity with cIL-10 and thereby offer a potentially simple strategy to target latently infected cells without off-target effects on cIL-10.
